# Microbial Contamination, an Increasing Threat to the Consumption of Fresh Fruits and Vegetables in Today's World

**DOI:** 10.1155/2020/3029295

**Published:** 2020-05-22

**Authors:** Gadafi Iddrisu Balali, Denis Dekugmen Yar, Vera Gobe Afua Dela, Priscilla Adjei-Kusi

**Affiliations:** ^1^Department of Theoretical and Applied Biology, Kwame Nkrumah University of Science and Technology, Kumasi, Ghana; ^2^Department of Science Education, University of Education, Winneba, Ghana; ^3^Kumasi Centre for Collaborative Research (KCCR), Kumasi, Ghana

## Abstract

Microbes are found all over the globe with some few exceptions, including sterilized surfaces. They include normal flora that is nonpathogenic, which contribute to the larger percentage, and pathogenic species which are few. Hence, the activities of humans cannot be completely separated from microbes. Thus, many pathogenic microbes have found their way into fresh fruits and vegetables which are a great source of a healthy diet for humans. The growing demand for fresh fruits and vegetables has necessitated larger production. The larger production of vegetables within the shortest possible time to meet the growing demand has placed them at a higher risk of contamination with the pathogenic microbes, making the safety of consumers uncertain. Study of sources of contamination and type of pathogenic etiological agents isolated from fresh fruits and vegetables includes *Bacillus cereus*, *Campylobacter jejuni*, *Clostridium botulinum*, *E. coli* O157: H7, *Listeria monocytogenes*, *Salmonella* spp.*, Shigella*, *Staphylococcus*, and *Vibrio cholera*. Several measures have proven to be effective in controlling contamination of microbes and they include the establishment of surveillance systems to monitor the production chain and thoroughly washing vegetables with vinegar water. Saltwater and other washing techniques are effective but caution should be taken to make sure one does not use one cycle of water for washing all vegetables. The consumption of fresh fruits and vegetables is still encouraged by this review but significant measures must be taken to check the safety of these products before consumption.

## 1. Introduction

The global production of fresh vegetables and fruits has increased by 30% over the last few years [1]. It has increased from 30 million tons to 60 million metric tons [2]. This increment has been gradual and hence the increase in exports is at pace with the growth of fruit and vegetable production worldwide [3]. However, the value of the European countries in the export of fruit and vegetable trade is gradually declining [4]. In comparison to other continents of the world, there are a lot of variations although both exports and imports in the rest of the continents are increasing at a faster rate. While the production and exports of fruits and vegetables in Asian countries have almost doubled in the last few years [5, 6], the growth of fresh fruits and vegetable production from Africa and American (Latin) countries is slower as compared to other continents.

Fresh vegetables and fruits play an important role in human nutrition due to their high nutrient content of vitamins, such as vitamins B, C, K, and minerals such as calcium, potassium, and magnesium, as well as dietary fibre [7]. Fresh fruits and vegetables provide a healthy and balanced diet and can prevent chronic diseases such as heart diseases, cancer, diabetics, and obesity including several micronutrient deficiencies especially in developing countries [8]. Vegetables consumed raw are increasingly being recognized as important vehicles for the transmission of human pathogens [9]. As fresh vegetables are eaten raw or slightly cooked to preserve the taste and their nutrient contents, this serves as a potential source of various food-borne infections and disease outbreaks [10]. While there is an increase in global consumption of fresh fruits and vegetables, this is greatly threatened by an upsurge of microbial contamination [11]. There is, however, a paucity of up-to-date knowledge on the epidemiology of microbial contamination, route, and sources of contamination of fruits and vegetables. This manuscript is, therefore, designed to review and synthesize existing literature to update the current knowledge gap and provide possible future technologies on food safety on fresh fruits and vegetables.

## 2. Vegetable Production Distribution

**Figure 1:**
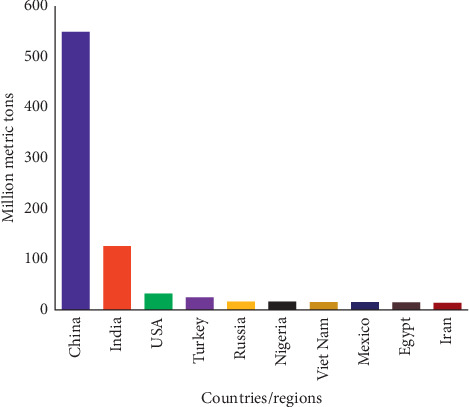
World ten leading producers of vegetables [3].

**Figure 2:**
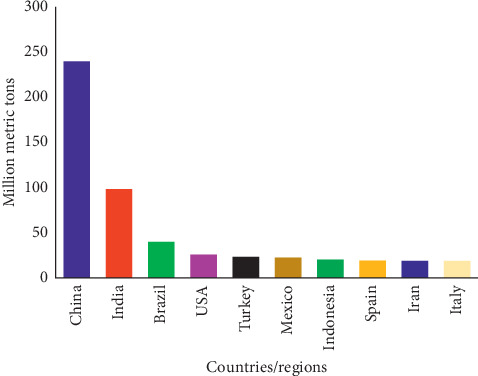
World ten leading producers of fruits [3].

The production of vegetables is distributed among the world's best 10 vegetable producing countries including China, India, Iran, Vietnam, Turkey, Russia, Nigeria, Egypt, Mexico, and the United States of America. These countries are distributed among four major continents in the world (Figures 1 and 2).

While countries in Africa were able to make it among the world top ten producers of vegetables, fruit production had a different pattern; thus, the distribution was among China, India, Brazil, USA, Turkey, Mexico, Indonesia, Spain, Iran, and Italy consisting of only three continents (Figure 2).

## 3. Methodology

A well-organized review of microbiological literature on microbial contamination of fresh fruits and vegetables was conducted. It focused specifically on major and minor issues on the topic including information having a bearing on the topic. Articles in high impact journals were downloaded and used for the study. We search for studies conducted using the following phrases: microbial contamination of vegetables, microbial contamination of fresh fruits, microbial contamination of fresh fruits and vegetables, guidelines for reducing microbial contamination of vegetables, and diseases commonly associated with microbial contamination of vegetables.

The reviewed articles had either experimental or nonexperimental designs in the study. Google Scholar, PubMed, Base-search.net, Science Direct, and Microsoft Academic were the search engines used to download the articles. Other sources of information included Institutional Repositories, Food and Agricultural Organization (FAO), United Nations (UN), and the World Health Organization (WHO). The key search terms that were used in the review were microbial contamination, bacterial contamination, fruits contaminants, vegetable contaminants, and food-borne illness. In total, four different types of searches were done in a systematic manner using inclusion and exclusion criteria and that of the search terms. With regard to the first search, articles identified in the specified databases and their complete copies obtained and deemed fit for inclusion criteria were considered for further review. Articles that did not meet the inclusion criteria stated above were excluded, as they were said to have been conducted on parasites population, were duplicates on other databases, or just dealt with only antiracial methods.

For search two, articles obtained through cited articles were looked at following the criteria used for search one; 11 articles were found but not all were included in the study. Search three was conducted one week after the first search. This was to capture any recently published articles.

Search four was done some months later with an increase in the number of search engines and other international bodies with factual information. Search four resulted in a total of about 1,782 articles, book chapters, and others. Some documents were downloaded two to four times due to the difference in search engines and titles used to upload documents. After carefully removing all duplicated documents, a total of 489 documents were obtained. Reading titles of the documents, an additional elimination was done. Also, a further reading of abstracts and whole documents and subjecting them to exclusion and inclusion criteria finally resulted in 42 documents. These included review papers, research articles, case studies, and case reports.

## 4. Microbial World

### 4.1. Microbes as an Inevitable Life Form

Microbes are essentially microscopic organisms which in one way or the other fit the description of bacteria, fungi, protozoa, viruses, and algae that are found almost everywhere on Earth [12, 13]. Most of these microbes serve as the beginning and end of complicated food chains from which all life forms depend on for survival and existence [14]. This makes them very important to humans [15] and other organisms [16]. Out of a total of about 100 trillion cells in the human body, about one-tenth of these cells are not real cells but microbes [14]. Viruses, bacteria, fungi, and other microorganisms account for trillions of cells in the human body. Some microorganisms are commensals, others are mutualistic, while some are infectious agents [17]. They all play significant roles in immunity and function in areas of modulation [18], metabolism, and drug interaction in the body [19].

#### 4.1.1. Bacteria

Bacteria are unicellular prokaryotic organisms medically classified based on their shapes [20]. These are spiral/curved bacteria, bacilli/cylindrical/rod-shaped bacteria, and cocci/spherical bacteria. Concerning the quantity of peptidoglycan in their cell walls, bacteria can be classified into two essential groups, namely, Gram-positive and Gram-negative [21]. While Gram-negative bacteria, such as *Escherichia coli* O157: H7, *Salmonella* spp., and *Proteus mirabilis* [22], are generally known to cause numerous diseases, *Bacillus cereus*, *Clostridium botulinum*, and *Clostridium perfringens* are typical Gram-positive bacteria responsible for causing intoxications in food [23, 24]. Numerous other bacteria cause infections and food spoilage; they include *Acinetobacter, Alcaligenes, Aeromonas, Flavobacterium, Arcobacter, Lactococcus, Pseudomonas, Serratia, Shigella, Listeria, Yersinia, Campylobacter, Citrobacter, Vibrio Enterobacter, Micrococcus, Enterococcus, Paenibacillus, Corynebacterium, Staphylococcus,* and *Weissella* [25].

Although some of these bacteria have been shown over time to cause harm, some bacteria are necessary for our daily lives and help in digestion, decomposition, and the production of food such as cheese, bread, and yoghurt, such as some strains of *Lactobacillus, Bifidobacterium*, *Erwinia,* and *Streptococcus*. *Lactobacillus bulgaricus* is well known throughout the world for the production of yoghurt [26]. Some industries also utilize *Streptococcus thermophiles* in producing yoghurt.

#### 4.1.2. Fungi

Moulds and Yeast are classified into the kingdom fungi. Moulds are filamentous multicellular characterized by cottony/fuzzy appearance on the surface of food [27]. Moulds require little moisture and survive in temperatures within 25–30 ^0^C and with low pH levels; they can grow tremendously on most grains and corns when stored in moist areas. Moulds such as *Camembert* and *Roquefort* are useful in the production of various foods and food products and the ripening of foods such as cheese. They are also useful in the production of feed and food and also serves as a catalyst (enzymes) in the manufacture of bread or citric acid used in soft drinks [27]. *Botrytis cinerea* is employed in the decomposing process of grape for the production of wine.

In the food industry, yeast is normally used to ferment sugars to CO_2_ and ethanol. *Saccharomyces carlsbergensis* which is an industrially grown form of yeast is used in the fermentation of most beers [28]. Yeasts can ferment sugars to ethanol and carbon dioxide and hence they are extensively in the food industry. The most commonly used yeast, the baker's yeast, is grown industrially. *Saccharomyces carlsbergensis* is most commonly used in the fermentation of most beers [29]. The other yeast strains of importance are *Schizosaccharomyces*, *Hanseniaspora, Candida, Zygosaccharomyces, Cryptococcus, Saccharomyces, Brettanomyces,* and *Debaryomyces* [30].

Some important microbes such as bacteriophage (phages) act as biocontrol to eliminate other harmful organisms in foods [31]. They are said to be highly specific with regard to the action of antibiotics. They do not infect humans and other normal flora including the gut flora. They generally help to decrease the prevalence of opportunistic infections.

#### 4.1.3. Viruses

Viruses are said to be obligate intracellular organisms that necessarily needs a live vulnerable host to establish infection and cannot proliferate in foods outside a living host. They are usually transmitted through fomites, water, food, and one person to the other through contact with an infected individual [32]. There are many food-borne viruses in existence; however, the most common ones include hepatitis A virus (HAV), astroviruses, adenoviruses serotypes 40 and 41, human noroviruses (HNoV), parvoviruses, hepatitis E virus (HEV), sapovirus, rotavirus (RV), coxsackievirus A and B, Aichi virus (AiV), enteroviruses, parvoviruses, and picornaviruses. Many pathogenic viruses are responsible for the highest causes of nonbacterial gastroenteritis [33].

**Figure 3:**
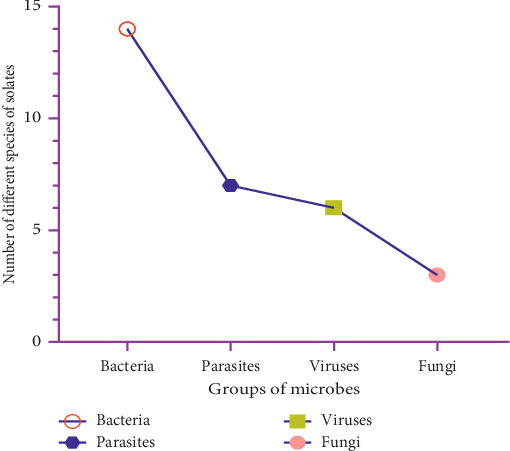
Groups of microbial species isolated from fresh produce.

Etiological agents responsible for causing diseases are distributed among most various groups of microbes including parasites.  Figure 3 provides a diagrammatic understanding of the major isolated etiological agents from fresh produce with regard to the various groups of microbes.

**Figure 4:**
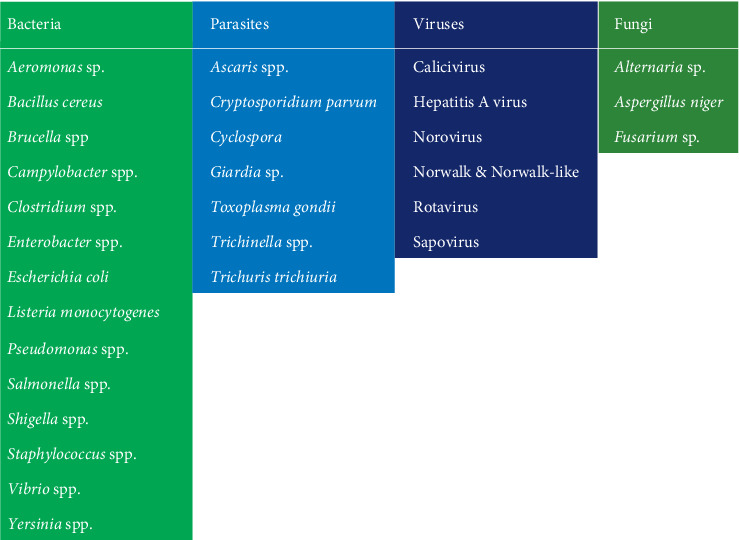
Typical etiological agents frequently isolated from fresh produce.

The increasing number of etiological agents (Figure 4) leads to an outbreak of diseases posing a major health threat to humans [34] and the world at large. This instigates the mind to ponder the sources of these diseases and a possible remedy. Significant knowledge of food-borne illnesses that are associated with freshly consumed fruits and vegetables and even the water used in the preparation of foods in towns and cities of many countries such as Ethiopia, Nigeria, Ghana, India, Brazil, China, UK, USA, Germany, Indonesia, and Iran is essential in combating the situation. Several studies have shown that food-borne illness has a strong association with microbial contamination [35–38].

Numerous studies revealed that outbreaks of diseases like typhoid fever, dysentery, diarrhoea, and even cholera are a result of the consumption of pathogenic microbes or their toxins, etc. [39]. This current study is, therefore, designed to review existing literature on microbial contamination of fresh fruits and vegetables. It will further access literature to establish the association between the identification of various pathogenic microbes and their attributes to food-borne disease outbreaks.

## 5. Global Consumption of Fresh Fruits and Vegetables

Dating back from 1986 to 1995, about 0.95% of vegetables per capita and 0.38% fresh fruits per capita consumption were recorded, with China leading with the highest consumption of fruits accounting for 6.4% [40]. The lowest consumption of fresh fruits and vegetables was recorded to be 0.19% of vegetables in Sub-Saharan Africa, and there was a decline in consumption rate in Africa and Near East Asian countries [40]. It has been revealed that the production of fresh produce such as fruits and vegetables has experienced a tremendous increment of about 94% from the year 1980 to 2004 [40]. This has been attributed to population growth globally.

**Figure 5:**
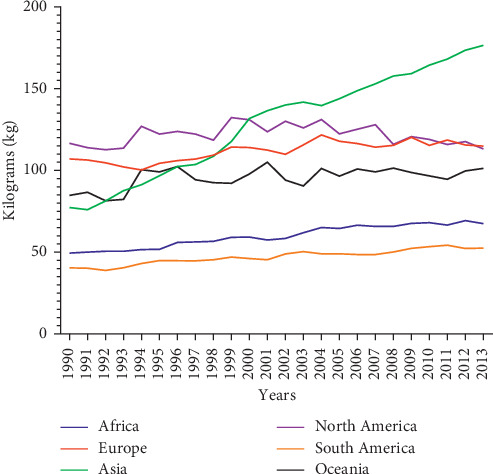
Continental vegetable consumption per capita [3].

Fruit and vegetable consumption shows different patterns in recent years. Asian countries recorded the highest rate of consumption, followed by Europe, Northern America, Oceania, and Africa [3]. Consumption rate in Europe was found to be slightly higher than that in Northern America which recorded a sharp decline in consumption per capita over the subsequent years. Oceania recorded a steady rise in consumption but was slightly lower when compared to Asia, Europe, and North America [3]. In Africa, the consumption rate over the last 23 years continues to rise but at a slower pace. This could be attributed to the increase in population. This is relatively slow with comparison to the other continents (Figure 5).

**Figure 6:**
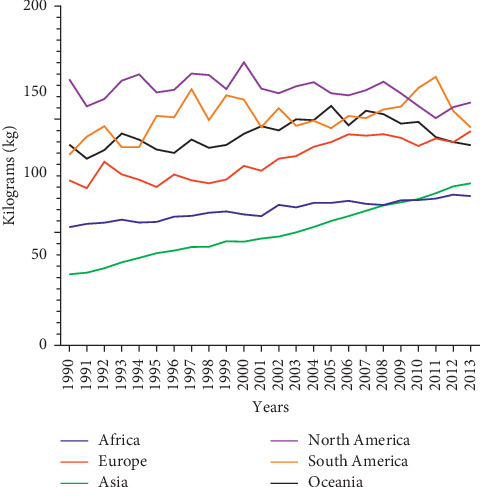
Continental fruit consumption per capita [3].

Fruit consumption over the last decade has seen a steady rise in consumption. Between 1990 and 2000, the rate of consumption increased slightly among most continents. North and South America, Oceania, and Europe experienced an uneven consumption rate. Asia and Africa, on the other hand, experienced a steady rise [3]. From 2001 to 2013, Africa and Asia consumption pattern as compared to Europe, North and South America, and Oceania continue to show steady increase (Figure 6).

**Figure 7:**
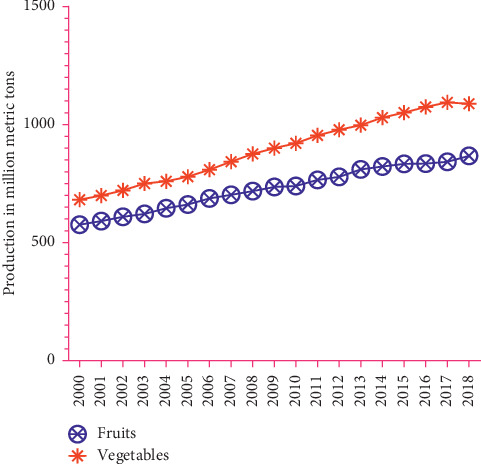
Trends in the global production volume of fresh vegetables and fruits.

The production of fruits and vegetables has increased significantly over the last few years. Between 2000 and 2010, the level of production of vegetables increases at a higher rate. It continued to increase gradually until 2018 when the production drops slightly [3]. Notwithstanding, fruit production increases tremendously during the last eighteen years. However, with regard to volume, vegetables recorded the highest production as compared to fruits (Figure 7).

Due to the increasing urban population and travelling of long distances to work in India, many people prefer to eat sprouts, fruits, and raw vegetables than any fast food because it is believed to be healthy [40]. The world health organization conducted a survey on vended food along the street which also established that fresh fruits and vegetables form about 86% of the total food market [40]. Countries such as America are best known to be major sources of fresh produce and it is significant to know that about 35% is obtained by importation. Based on the numerous nutrients provided by fresh fruits and vegetables in our daily diet, these must be free from contamination [41].

Globally, fresh fruits and vegetables contribute significantly to the food market and hence their safety is also a global issue [40]. As China and India are larger producers of fresh fruits and vegetables, they are also faced with the problem of contamination. The safety of fresh produce is a concern for not only the consumers and importers but also the producers. In Africa, the consumption of cabbages, onions, tomatoes, and other vegetables increases because they are readily available in the market, and hence accessible, convenient, and less costly as compared to that of fruits including apples, grapes, and others [42]. The higher demand for fresh fruits and vegetables has placed pressure on the chain of production right from farmers to the sellers and finally consumers [43]. Farmers in Africa that produce a lot of vegetables and fruits for local consumption are faced with several threats such as poor water quality, insects' infection, and hence the necessitation of investigation into microbial contamination [44].

In the process, care for safety reduces and many vegetables become contaminated with pathogens. Yet, there is not much-published data on the knowledge of farmers and retailers of fresh fruits and vegetable contamination with microbes and its implication on human health [40].

## 6. Epidemiology of Microbial Contamination

The greater concern for human health geared towards the promotion of a healthier lifestyle by public health promotion movements in both developed and developing countries has triggered a tremendous increase in the consumption of fresh vegetables and fruits. In some developed nations, the production of fresh fruits and vegetables has increased tremendously with an increase in the importation and even with improvements sustaining the eminence of fresh produce in the USA [45]. Following the consumption trend for fresh fruits and vegetables, there has been a tremendous increment of about 25% of weight following person consumption during the years of 1997–1999 compared with that of 1977–1979 [46], and also between 1999 and 2010, changes in prices and total food expenditure drove most food-purchasing patterns in the United States [47]. Nevertheless, the consumption of fresh fruits and vegetables globally has increased tremendously from 2011 to 2018. Hence, according to the Centres for Disease Control and Prevention (CDC), there has been an increase in the rate of contamination of fresh fruits and vegetables in recent years [48].

## 7. Major Sources of Contamination

Fruits and vegetables may be contaminated at any point in time during the production chain. Sources of contamination can be grouped into two broader groups, namely, preharvest and postharvest sources of contamination [49]. With regard to preharvest sources of contamination, studies have shown that the soil in which fruits and vegetables are cultivated may be a source, and also water used for irrigation, water used to apply insecticides and fungicides, faeces, dust, improperly composted manure, and finally human interaction with these vegetables at various points during the production period.

Dry season farming and its associated microbial contamination of fresh fruits and vegetables in poor regions of the world need to be researched [44]. The use of irrigation method of farming during the dry season is a major practice in Africa. However, in Sub-Saharan Africa, many vegetable crops are produced in fresh forms using the irrigation method. They mostly use dirty water or wastewater in the watering of the crops [44]. This gives some of the microbe's opportunity to contaminate the plants and subsequently the consumers. Similarly, many farmers use this same water in the application of fungicides and weedicides which can also result in contamination with coliforms [44]. Moreover, the application of poultry manure and other incomplete compost to the crops can also result in contamination with enteric bacteria in faeces. Research has also shown that some pathogens including *Escherichia coli* O157: H7, *Listeria monocytogenes*, and *Salmonella* spp. have been isolated from animal faeces including poultry and cattle [43, 50]. It has been confirmed a few years ago that *E. coli* O_157_: H_7_ can be transmitted to lettuce through the soil and irrigation water and can persist throughout the life cycle of the plant and can further be transmitted to those who consume the crop [39]. Another research has shown that there is an association between salmonellae, stems, and leaves of tomatoes grown hydroponically in inoculated solutions. This situation could be minimized by understanding the sources and managing them properly using methods like changing conditions, disinfection of contaminated waters before use, etc. not only during farming but also during processing after harvest, known as postharvest.

Postharvest sources of contamination include faeces, harvesting equipment, human handling, insects, wild and domestic animals, methods of transportation, processing equipment dust, and rinse water [49]. The use of the pond and river water in washing fresh produce places them at a higher risk of contamination since these waters are most likely to contain some pathogenic microbes [51]. These same people handle the vegetables and most of them are already infected with these pathogens serving as fomites and the storage of these products is mostly done in contaminated places.

In the traditional trading of fresh fruits and vegetables, several studies have established that watering, washing, handling, and storage are major sources of numerous contaminations with microbes even though it is difficult to establish a precise link between the contamination of these fruits and vegetables with the outbreak of food-borne illnesses [42, 44, 52, 53]. In Africa, vegetables are mostly washed easily to obtain water sources including rivers and ponds that are near to the production or selling site [42]. Containers used in washing vegetables by farmers as well as fruits and vegetable vendors are not mostly washed after use, and even if washed, the water is used for several cycles allowing for cross-contamination of microbes with the recently washed ones since they are put in the same water as the first cycle [42]. Washing containers should be disinfected before use and after use to ensure the safety and prevention of microbial contamination.

Fruits like mangoes are mostly handled with bare hands during harvesting, packaging, and distribution; hence, it has been established that many mangoes in the country are contaminated before they are sold, which, when not properly washed, will result in food-borne illnesses [54]. Bananas are also largely produced worldwide and sold both in the international market mostly by developed countries and locally mostly by underdeveloped nations. While developed countries adhered to many food standards such as the Codex Alimentarius Standards, developing countries do otherwise. For example, there have been issues of strict labelling of food products and categorizations into organic and nonorganic foods in developed countries. However, in underdeveloped nations such as Africa, the sellers usually buy them from the farmers unripe and then ripen them with chemicals and this gives way for microbial invasion resulting in contamination [55]. There are several pathogenic microorganisms associated with fresh fruits and vegetables and hence insight into the various kinds and species is very important.

**Figure 8:**
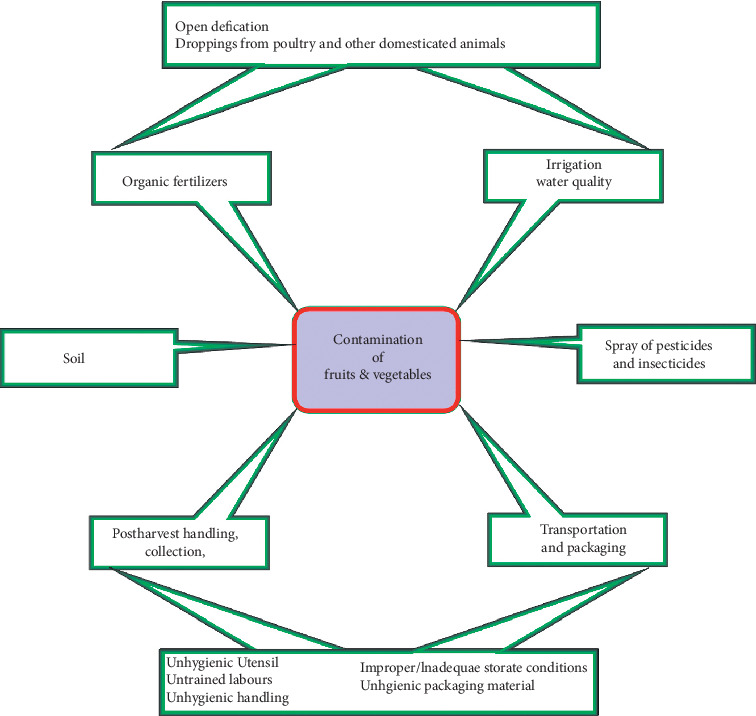
Factors contributing to the contamination of fruits and vegetables.

Based on the information available, it is clear that there is not much information on the sources of contamination of our water bodies and their relationship with vegetable farming with its link to an infection. Faecal microbes like *E. coli* isolated from various vegetables have been established to be a result of faecal contamination. But there is no precise study to elucidate the source of *E. coli*, whether from open defecation or dislodged toilets (Figure 8).

## 8. Routes and Chain of Contamination

Origins of pathogenic microbes that usually contaminate fresh fruits and vegetables can be traced to the human pathogens, poultry, and other domestic animals' microbes including soil microbes and other activities that create a way for microbial colonization of fresh fruits and vegetable surfaces (Figure 8).

## 9. Threats to Fresh Vegetable and Fruit Consumption

Several pathogenic bacterial species are primarily responsible for the contamination of fruits and vegetables as having been evidenced by the isolation of these species from various fruits and vegetables (Table 1), such as *Escherichia coli* O_157_: H_7_, *Listeria monocytogenes*, *Salmonella* spp., and others from many fruits and vegetables including lettuce, cabbage, and cucumbers [79]. Also, in the year 2011, Germany recorded one of the highest outbreak of EAEC O104, amounting to over 2220 cases [62]. Another incident memorable is the recent outbreak of *Listeria monocytogenes* in South Africa [57].

## 10. Contamination and Food-Borne Illness Outbreaks

The isolation and linkage of transmission of food-borne illness by various pathogenic etiological agents from an animal source are proven by scientists [80]. Though no gigantic clinical studies have been able to directly link isolation of pathogenic helminths, bacteria, viruses, and protozoa to the outbreak of food-borne illnesses associated with the consumption of fresh fruits and vegetables, there has recently been an increasing threat with the levels of pathogenic microbes found on fresh fruits and vegetables consumed locally and exported to other neighbouring countries [39]. These trends are thought to have a link with the tremendous increase in the demand and consumption of fresh fruits and vegetables which are produced locally in various developing countries in Africa [81, 82]. Environmental and economic conditions of both producers and market women in the country make this fresh produce vulnerable to human pathogens. It has been estimated that about 12% of food-borne illnesses in the previous years, somewhere around the 1990s, were strongly linked to the consumption of fresh produce including fruits and vegetables [40].

In most developing countries, the poverty of farmers and retailers is a major issue contributing to less or no disinfection of fresh produce before marketing [83]. One washing container with water is used to wash several vegetables leading to contamination instead of disinfection [41]. The higher the number of cycles of washing, the more vulnerable the later cycles are prone to contamination. Also, recent outbreaks of food-borne illnesses in Ghana were attributed to the consumption of faecal pathogens including *Vibrio cholera*, *Salmonella typhi*, and others [84]. Bacteria and viruses are said to be the common causes of food-borne illnesses and attributed to the consumption of fresh fruits and vegetables that are contaminated with various etiological agents (Table 2). About 48 out of 94 outbreaks are linked to viruses as compared to 20 out of 102 linked to bacterial contaminations [40]. Hence, it is clear that the outbreaks of food-borne illnesses are mostly of viral origin. Temperature and humidity are factors that determine the survival of pathogens on fresh fruits and vegetables, especially viruses [49]. Recently, there has been a significant increase in technology to control or optimize the microbiological safety of fresh fruits and vegetables [88], and hence their consumption must still be encouraged as they form an important part of a healthy diet.

## 11. The Worldwide Scenario of Pathogenic Microbes Isolated from Fresh Fruits and Vegetables

In October 2006, a study was conducted across 26 states in the USA and Canada regarding an outbreak of *E. coli* O157: H7 which was found to be associated with the consumption of spinach. Of 199 persons infected with the pathogen, three deaths were recorded and reported to the Centre for Disease Control and Prevention (CDC). And out of the cases reported, about 16% developed acute renal failure while 51% of the said cases were hospitalized [89]. Another outbreak of *E. coli* that dazed the world led to 50 deaths and hospitalizations of about 4,000 patients in about 16 countries [40]. This caused scientists over the world to ponder over the outbreak and possible solutions for future outbreaks [40]. Not only *E. coli* but also *L. monocytogenes* outbreaks triggered the scientific community [40].

Meanwhile, the busy nature of today's world has propelled the people to change their lifestyle because there is not much time for cooking, and, therefore, they need to buy food almost all the time. In places like Ghana, many people prefer eating fast food to traditional ones like “fufu” and “banku” and other foods which will take time to serve [90]. So the patronization of fresh fruits and vegetables is seen as less time-consuming. A study conducted in Santiago Chile which took about six years to complete revealed that raw-eaten-vegetables were contaminated with *L. monocytogenes* which was the first report of its kind in Chile specifically of the said pathogen [91]. Lack of specific killing agents for most food-borne pathogens and remedial steps to control them before consumption of fresh fruits and vegetables is a problem for the fresh fruits and vegetable industries [92].

Since there is no bactericidal or killing agent for combating contaminations of spinach and lettuce with enteric bacterial pathogens such as *E. coli* and *Salmonella* spp., enterohemorrhagic during the harvesting, processing, and packing procedures, the pathogens tend to survive even better and stand the chance of human infection [39]. One particularly persistent pathogenic organism that can survive under harsh conditions including low temperatures (freezing conditions), low pH, and even high salt concentrations is *Listeria. Listeria* causes listeriosis, an uncommon disease but dangerous. Nonetheless, listeriosis accounts for about 30% death rate in comparison with other food-borne pathogenic microbes [93]. Fresh fruits and vegetables mostly have a higher risk of contamination during preparation, distribution, and storage [45, 49]. As a result, developing countries like Ghana where fresh fruits and vegetables are mostly produced by poor farmers who have little or no knowledge of food-borne illnesses will continue to face the challenge of contaminations [54].

## 12. Food Safety Technologies and Policy

### 12.1. Measures to Manage Microbial Contamination of Fresh Fruits and Vegetables

Tracing the production chain of fresh fruits and vegetables, and considering the sources of infection, there is currently no effective antimicrobial treatment that can be used at any step in the production chain (from planting to consumption) that effectively and efficiently remove pathogenic microbes [43]. This, therefore, means that contamination at any given time during the production chain can present and probably multiply on the final product that is ready for consumption. There are several methods employed in controlling or trying to control the contamination of pathogenic microbes associated with fresh fruits and vegetables. These include washing and rinsing vegetables under running water which is known to increase the shelf-life of some fresh fruits and vegetables through decreasing or removing microbes on their surfaces [94]. Nevertheless, only a few pathogenic microbes may comply with this method of treatment while a good number of them will persist. Other treatment methods including disinfection which can increase the efficiency of removal of microbes for about a hundred times as much as the previous method of treatment. Also, chemical treatments directed to the entire and cut fruit or vegetable naturally will not decrease populations of pathogenic microbes by more than 2 to 3 log_10_ CFU/g World Health Organization [94].

Ozone is an effective and efficient method of pathogenic microbial elimination from fresh vegetables like lettuce [40]. It is, however, stated that its effectiveness is dependent on several factors including the residual ozone in the medium and quantity of ozone used [95]. Other factors such as humidity, pH, temperature, and additives (surfactants, sugars, etc.), including the quality of organic matter enclosing the cell in a study, showed that an application of 2 ppm ozonized water treatment to leaf lettuce for about 2 minutes is found to be effective in ozone disinfection in the lettuce [40]. This subsequently helps in decreasing microbial load and leaving a good sensory quality during cold storage. The method was also found to be more effective than chlorine and organic acid methods of treatments in maintaining the sensory quality of lettuce during nine-day storage [95].

Phage control of fruit and vegetable contamination has recently gain stands and has been employed in the control of numerous pathogens in food products such as carrots and tomato [96, 97].

## 13. Conclusion

Microbes are associated with every single matter that exists except sterilized bodies or places. This means that microbes are inevitable life forms on Earth because they are almost everywhere. Their variation in forms gives them the ability to survive in almost all kinds of environments. Since they are found in all unsterilized environments, then it is not surprising that they are found on fresh fruits and vegetables.

Fresh fruits and vegetables are used all over the globe with an ever-increasing demand as population increases. Today's endless stream of activities is also causing many people to be busy all times, thus affecting their diets and triggering a switch to the consumption of fruits and vegetables to save time, but the main contributor to the greater consumption and utilization of vegetables globally is their nutritional content. Fresh fruits and vegetables have become known and accepted as a healthy diet that helps correct many food imbalances in the body. The higher demand for fresh fruits and vegetables has made producers look for cheap and fast methods of production and hence less concern about their safety.

Yet, at the same time, today's society faces a greater challenge in the consumption of fresh fruits and vegetables as pathogenic microbes have been isolated from these products, indicating their presence. Hence, several outbreaks of food-borne illnesses have been associated with the consumption of fresh fruits and vegetables. Therefore, the need for appropriate measures to optimize contamination conditions for fresh fruits and vegetables has now become a global concern. And identifying and dealing with sources of contamination is the first step in the process.

Again, farmers have to use the irrigation method in Ghana to cultivate cabbage in the dry season and most at times with contaminated waters. The market women in Ghana also buy from farmers and do not wash with disinfectants and sell at the market. Consumers who buy these fresh fruits do not also take appropriate measures to disinfect them or wash thoroughly; hence, vegetables are still contaminated with various pathogens. This places the consumers at a higher risk of being infected. There is, therefore, the need for appropriate measures to be taken to minimize contamination of fresh fruits and vegetables throughout the production table.

Appropriate surveillance systems should be set up throughout the production table to also monitor when, where, and what is to be done when there is contamination. Washing vegetables thoroughly with vinegar water, saltwater, and other washing techniques are effective but caution should be taken to make sure one does not use one cycle of water for all vegetables. This might cause further contamination of previous cycles. So, the consumption of fresh fruits and vegetables is still encouraged by this review but significant measures must be taken to check the safety of these products before consumption.

## 14. Recommendations

Professionals and other people including farmers and market women involved in the food production industry ranging from production to the market should be made aware of the potential risk associated with various practices and possible chances of contamination. They should be educated to gain sufficient knowledge on the source of etiological agents responsible for the contamination and their resultant diseases.

## Figures and Tables

**Table 1:** Country profile of etiological agent's isolation.

Country	Product	Pathogen	No. of cases/isolates	Reference/year
*Africa*				
Nigeria	Fresh vegetables and meat	*Listeria monocytogenes*	—	[56]
South Africa	Fresh produce	*Listeria monocytogenes*	—	[57]
Egypt	Orange juice	Hepatitis A	351	[58]
Kenya	Maize	Hepatitis B and Aflatoxin	317	[59]
Rwanda	—	*Salmonella typhimurium*	78	[60]

*Europe*				
Sweden	Salads	*Listeria monocytogenes*	51	[61]
Germany	Fresh produce	*Escherichia coli* O104: H4	2229 cases	[62]
	Strawberries	Norovirus	11,000	[63, 64]
Turkey	Green leaf lettuces, cos lettuces, iceberg lettuces, spinach, and carrot	*Salmonellosis* and *Campylobacteriosis*	—	[65]
Denmark	Carrot and lettuce	*Cryptosporidium hominis*	78	[66]
Finland	Raspberries	Norovirus	200	[67]
Norway	Iceberg lettuce	*Shigella sonnei*	110	[68]
United Kingdom	Basil	*Salmonella spp.*	32	[69]

*Asia*				
China	Sprouts	*Salmonella enteritidis*	—	[70]
China	Rice	*Salmonella enteritidis*	197	[71]
Japan	Beans, cucumber and sprout	*Salmonella spp. Escherichia coli O157: H7, Staphylococcus aureus, Campylobacter* spp., and *Listeria monocytogenes*	—	[72]
India	Sprouts	*Escherichia coli*	923	[73]
Southeast Asian cities	Waters	*Escherichia coli*	—	[74]
Singapore	Bean sprouts and fresh-cut salads	*Escherichia coli O157: H7* and *Salmonella spp*., yeasts and moulds	—	[75]

*North and South America*				
Canada	Mango	*Cyclospora cayetanensis*	17	[76]
Mexico	Cantaloupes	*Salmonella spp.*	—	[48]
USA	Green onions	Hepatitis A virus	111	[77]
USA	Tomatoes/peppers	*Salmonella*	1442	[69]
USA	Cantaloupe	*L. monocytogenes*	147	[48]

*Oceania*				
New Zealand	Blueberries	Hepatitis A	81	[78]
Australia	Sprouts (Alfalfa)	*Salmonella*	100	[69]
Australia	Lettuce	*S. anatum*	144	[72]

**Table 2:** Some etiological agents isolated from various fruits and vegetables.

Pathogen	Product	Resultant complication or disease caused
*Aeromonas*	Alfalfa sprouts, asparagus, broccoli, cauliflower, celery, lettuce, pepper, and spinach	Opportunistic systemic disease in immunocompromised patients
*Bacillus cereus*	Alfalfa sprouts, cress sprouts, cucumbers, mustard sprouts, and soybean sprouts	Food poisoning
*Campylobacter jejuni*	Green onions, lettuce, mushroom, potato, parsley, pepper, and spinach	Food poisoning
*Clostridium botulinum*	Cabbage, mushrooms, and pepper	Botulism
*E. coli* O157: H7	Alfalfa sprouts, apple juice, cabbage, celery, cilantro, coriander, cress sprouts, and lettuce	Bloody diarrhoea and abdominal cramps
*Listeria monocytogenes*	Bean sprouts, cabbage, chicory, cucumber, eggplant, lettuce, mushrooms, potatoes, radish, salad vegetables, and tomato	Listeriosis
*Salmonella* spp.	Alfalfa sprouts, artichokes, beet leaves, celery, cabbage, cantaloupe, cauliflower, chili, cilantro, eggplant, endive, fennel, green onions, lettuce, mung bean sprouts, mustard cress, orange juice, parsley, pepper, salad greens, spinach, strawberries, tomato, and watermelon	Typhoid, salmonellosis, etc.
*Shigella*	Celery, cantaloupe, lettuce, parsley, and scallions	Shigellosis
*Staphylococcus*	Alfalfa sprouts, carrot, lettuce, onions sprouts, parsley, and radish	Food poisoning
*Vibrio cholerae*	Cabbage, coconut milk, and lettuce	Cholera
STEC	Salads	Diarrheal
*Hepatitis A virus (HAV)*	Blueberries	Hepatitis A
*Cyclospora cayetanensis*	Mango	Cyclosporiasis
*Fasciola hepatica*	Raw watercress	Worm infection
*Helicobacter pylori*	Basil, spinach, salad, parsley, leek, and radish	Gastritis, peptic ulcer disease, and certain types of stomach cancer

(Atapoor et al. [85]; McDaniel and Jadeja, [86]; Yeni, Yavaş et al. [87]).

## Data Availability

All data used for the study have been included in the article.
